# Effect of Sex and Age on Nutritional Content in Wild Axis Deer (*Axis axis* Erx.) Meat

**DOI:** 10.3390/ani10091560

**Published:** 2020-09-02

**Authors:** Nikolina Kelava Ugarković, Miljenko Konjačić, Zvonimir Prpić, Kristijan Tomljanović, Damir Ugarković

**Affiliations:** 1 Department of Animal Science and Technology, University of Zagreb Faculty of Agriculture, Svetošimunska Cesta 25, 10002 Zagreb, Croatia; mkonjacic@agr.hr (M.K.); zprpic@agr.hr (Z.P.); 2 Institute of Forest Protection and Wildlife Management, University of Zagreb Faculty of Forestry, Svetošimunska Cesta 25, 10002 Zagreb, Croatia; tomljanovic@sumfak.unizg.hr; 3 Institute of Forest Ecology and Silviculture, University of Zagreb Faculty of Forestry, Svetošimunska Cesta 25, 10002 Zagreb, Croatia; dugarkovic@sumfak.unizg.hr

**Keywords:** meat, minerals, amino acids, fatty acids, chemical composition, axis deer

## Abstract

**Simple Summary:**

Game meat is perceived as more nutritious than meat originating from domestic farm-ranged animal species. However, meat composition is affected by numerous factors, and differences between game species can be found. The aim of this study is to examine the effect of sex and age on the content of macro- and micro-nutrients in meat from axis deer. Sixteen animals were hunt-harvested and assigned to groups according to sex and age. Samples of *m. longissumus thoracis* were examined to determine proximate chemical, fatty acid, amino acid and mineral composition. Minor differences were found in the analysed traits between sex and age groups. Regardless of sex and age, axis deer meat is characterised as having a high protein and low-fat content, favourable fatty acid composition and ratios. It is a good source of essential amino acids and micro-minerals. As data regarding axis deer meat are limited, the results of this study are a valuable contribution to describing the quality and nutritional composition of meat of different deer species. Axis deer meat can be recommended as a healthier substitute to red meat.

**Abstract:**

The aim of this study is to examine the effect of sex and age on proximate chemical, fatty acid, amino acid and mineral content of axis deer (*Axis axis* Erx.) meat. Sixteen (*n* = 16) animals were hunt-harvested and assigned to groups according sex and age (sub-adult and adult). All analyses were made on *m. longissimus thoracis* sampled between the 9th and 13th ribs. Minor differences in nutritional composition of axis deer meat were found between analysed sex and age groups. Axis deer meat has a high protein (22.8%) and low fat (1.39%) content. Saturated fatty acids accounted for 44.97% and polyunsaturated for 29.66% of the total fatty acids. Ratios of fatty acids were within the recommended values. Glutamic and aspartic acid were the most abundant non-essential, and lysine and leucine the most common essential amino acids. The ratio of essential to non-essential amino acids was <1. Potassium and phosphorous were the dominant macro-minerals, while iron and zinc were the dominant micro-minerals. The results of this study show that regardless of sex or age, axis deer meat can be considered a good source of basic macro- and micro-nutrients, and can be recommended as a substitute for red meat from domestic animals.

## 1. Introduction

Numerous wild animal species are harvested across the world resulting in large quantities of game meat. In the period from 2000 to 2012, The Food and Agriculture Organization estimated that the production of game meat increased from some 1.59 million tons to 1.99 million tons [1]. This can be attributed to the increase in the human population and the need to find additional food sources in developing countries, but also to meet special consumer demands in developed countries. Game meat is considered a delicacy product, specific in taste and flavour and more nutritious than meat originating from domestic farm-ranged animal species [2,3].

Game meat is perceived as an organic product, harvested from wild-living animals, free of steroids, drugs and antibiotics. Increased interest in game meat consumption in developed countries also arises from increasing public awareness of poor animal welfare and environment pollution arising from conventional meat production [1]. The nutritional composition and quality of meat from different wild and farmed deer species has been the subject of numerous studies. Comparative studies of meat nutritional composition between two or more deer species (red, fallow and roe deer were presented by [3,4,5,6]. Effect of sex and/or age on meat quality was reported by Volpelli et al. [7], Cygan-Szczegielniak and Janicki [8], Daszkiewicz et al. [9], Dannenberg et al. [10], Piaskowska et al. [11], Švrčula et al. [12], Lorenzo et al. [13]. Bureš et al. [14] and Okuskhanova et al. [15] presented the results of quality attributes and composition of meat from deer species (red and fallow deer) and domestic breeds (Angus and Holstein; cattle and horse). Differences in wild and farm-raised deer meat quality have also been analysed [16,17,18,19,20]. The general conclusion of these studies was that deer meat is a low-fat and good source of unsaturated fatty acids, essential amino acids and minerals. Thus, it represents a good substitute for red meat from domestic animal breeds.

In addition to chemical composition, overall game meat quality is also determined by other factors, such as sensory attributes (aroma, flavour, taste) and harvesting methods (animal welfare) [21,22,23]. Game meat is characterised by a specific, intense taste and smell that is not found in meats of domestic animals [21,22]. Despite the favourable nutritional value, this makes some consumers (especially women and younger population) reluctant to choose game over domestic animal meat [22]. Although hunting for food may be of vital importance in some parts of world (Africa, Asia), in most parts of Europe and America this is not the case [23]. For some consumers, hunting is seen as a threat to animal welfare, especially if certain hunting methods are applied (hunting in fenced area, hunting with dogs, etc.). Hunting for sport or as hobby does not fit in with the ethical standards of modern consumer and wild game species are not perceived as a vital food source [23].

Deer are one of the most harvested wild species hunted either for food purposes or trophies. In addition to free-living deer species, numerous farms with organised and planned deer production can be found worldwide [14,16]. Axis deer (*Axis axis* Erx.), known as chital or spotted deer, is one such deer species that can be found both wild and farmed around the world. Axis deer lives wild in Australia, occurs in Armenia, the Andaman Islands, Pakistan, Papua, New Guinea, Australia, the United States (California, Texas, Hawaii) and South America (Brazil, Uruguay, Argentina) [24,25]. In Europe, the only successful introductions were to a few Adriatic Islands in Croatia resulting in three free-range populations on the Brijuni Islands, on the island of Rab and on the island of Dugi Otok [26]. According to the International Union for Conservation of Nature (IUCN) Red List of Threatened Species [27], axis deer has been categories as a species of ‘least concern’ (LC) and in some parts of the world (Hawaii, USA) it has been treated as an invasive species because of its ability to reproduce quickly and its lack of natural predators [28]. In these situations, when a non-native game species outnumbers indigenous wild game species and exceeds the capacities of the native habitat, hunting is the most effective and cost-efficient tool in wildlife management [29]. In Croatia, axis deer is listed as a hunting game species and is managed under the hunting legislation.

Axis deer (*Axis axis* Erx.) in Croatia was introduced in 1911 on the Brijuni Islands [26]. The exact origin of the imported axis deer is unknown, though they are believed to have originated from Germany [25]. Further attempts to introduce axis deer in continental Croatia failed. Nowadays, the axis deer population in Croatia is about 300 individuals, predominantly (50% of the population) on the Brijuni Islands which are protected as a national park and the axis deer there are not managed for hunting purposes. The remainder of the axis deer population in Croatia is situated on the island of Rab (30%) and the island of Dugi Otok (20%) [25]. On both islands, axis deer is primarily managed for hunting purposes (prized antler trophies), while meat is a by-product.

Axis venison has been considered to be among the most flavourful venisons in the world [30,31], described as having the mildest flavour and being the most tender of the commercially marketed venison. Most hunters in North America who harvest different deer species consider axis meat to be the best-tasting game meat [32]. There are no reports of the nutritional composition of axis deer meat harvested from wild or farm-ranged animals.

As axis deer is a rare and introduced species in the EU-Mediterranean region, the aim of this study is to examine the effect of sex and age on proximate chemical, fatty acid, amino acid and mineral composition content in wild axis deer (*Axis axis* Erx.) meat originating from the northeastern Adriatic island of Rab.

## 2. Materials and Methods

This study was conducted in accordance with the guidelines of the European Union Directive 2010/63/EU [33] and Croatian legislation (Animal Protection Act, Official Gazette 102/17; Regulation on the protection of animals used for scientific purposes, Official Gazette 55/13), and was approved by the Bioethical Committee for the Protection and Welfare of Animals of the University of Zagreb Faculty of Agriculture (Croatia) (Class: 114-04/20-03/08; Ref. 251-71-29-02/19-20-2, 14-07-2020).

### 2.1. Study Area, Animals and Sampling

**Figure 1:**
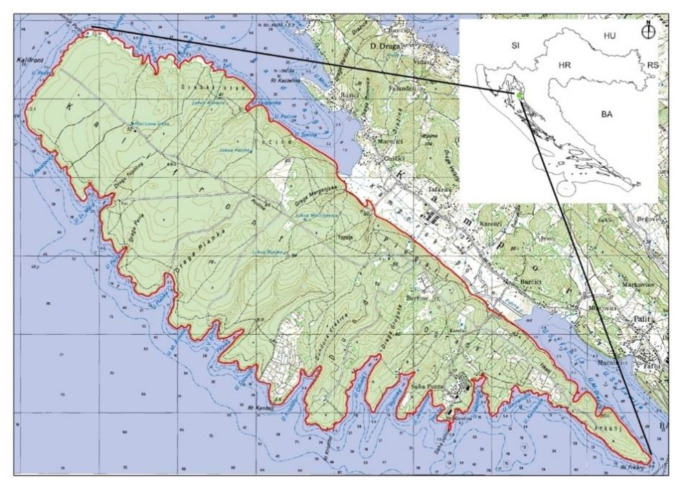
Map of the study area.

The study included 16 axis deer (eight male and eight female) originating from the island of Rab situated in the northeastern Adriatic Sea (44°47′24″ N, 14_4001000E). Introduction of axis deer to the island (in the forested area Kalifront-Topolje) occurred on 15 March 1974 when eight (6 females and two males) axis deer were transported from the Brijuni Islands [34]. Today, the axis deer population on the island Rab is about 80 individuals inhabiting 1351 ha of the open state hunting area Kalifront-Rab [35] (Figure 1). The hunting area in managed by University of Zagreb Faculty of Forestry, Croatia.

The axis deer diet in the study area is predominantly based on naturally available plant species. In the study area, axis deer co-occurs with more numerous mouflon population (*Ovis ammon musimon* Pall.) and both species are intermediate feeders [36,37]. Accordingly, previous studies of plant categories and plant species present in mouflon diet and analyses of mouflon rumen content [37] are also applicable to axis deer. The Eu-Mediterranean climate zone of the island results in less seasonal changes of forest vegetation [38]. Supplement feeding includes concentrate (corn, mixture: oat, alfalfa and ground corn), and hay. About 40 kg/animal of concentrate is evenly distributed through the year, and about 25 kg/animal of hay is provided dominantly during winter and summer when short-term extreme climate conditions can occur. Corn is distributed by automatic feeders, while mixture and hay are distributed at eight feeding places across the hunting area. About 2 ha of cultivated forage areas with alfalfa and grass-clover mixture is available. Supplement feeding of wild game in the Republic of Croatia is regulated by the legislation, though exact amounts are not stipulated.

Animals were hunted from 1 February 2018 to 28 February 2019 in accordance with the legislation of the Republic of Croatia [39]. Immediately after shooting, animals (*n* = 16) were exsanguinated and transported to the receiving plant situated in the Kalifront hunting area. In the receiving plant, carcasses were weighed, eviscerated, decapitated (atlanto-occipital junction) and skinned. Carcasses were then left to chill at +4 °C until 24 h post-mortem. The muscle *longissimus thoracis* (LT) was dissected from the 9th to the 13th rib on the right side of carcass, vacuum packed and stored at a temperature of −20 °C until analysis. For each individual, age was estimated according to phenotype characteristics and tooth evaluation [40] and two age groups were formed: sub-adult (24–47 months old; eight individuals including five females and three males) and adult (>48 months old; eight individuals including three females and five males).

### 2.2. Chemical Analyses

Prior to chemical analyses, samples were thawed, external fat was removed, minced, mixed and homogenised. Dry matter in axis meat samples was determined by adding 5 g of sample to an aluminium container containing approx. 2–3 g quartz sand. To this, 2 mL 96% ethanol was added and the sample was scrubbed with the sand. The resulting pulp was dried at 103 °C for 4 h. The sample was then cooled in a desiccator and weighed [41].

Fat content in meat samples was determined by measuring 5 g sample in a 400 mL beaker. Some pumice stone was added with 50 mL 4 M HCl solution. The beaker was placed on a hot plate and boiled for 1 h, then filtered on filter paper, washed with water and left to stand overnight to dry. The filter paper was placed in an extraction thimble and extracted with hexane for 4 h. After evaporation of the hexane, the sample was dried in a 98 °C oven and weighed [42].

To determine the protein content, a 1 g sample was measured in a glass tube for Kjeldahl analysis. Concentrated sulfuric acid (13 mL) was added and the sample was digested at 420 °C for 1 h. After cooling, the protein content was measured according to the Kjeldahl method, with a FOSS Kjeltec 8400 (Hilleroed, Denmark) protein analyser [43].

To determine the ash content, a 5 g sample was measured in a crucible and pre-dried in a 100 °C oven and then turned into ash in a furnace at 550 °C for 4 h. After cooling, the sample was weighed [44].

Fatty acid methyl esters (FAMEs) in axis *longissimus dorsi* samples were determined by gas chromatography according to [45]. Methylation of the samples was performed using a saturated sodium-chloride solution. Fatty acid methyl esters were quantified using a Shimadzu GC2010 gas chromatograph equipped with a silica capillary column, CP-Sill 88 (100 m length, 0.25 mm wall coated open tubular-WCOT, 0.2 µm, Varian, Santa Clara, CA, USA). The temperature program ranged from 130 °C to 202 °C. The injector and detector were both maintained at 270 °C. Fatty acids were identified by comparing relative FAME peak retention times of samples and fatty methyl ester standards from Supelco (Supelco 37 Component Fame Mix 47885-U, Sigma Aldrich, St.Louis, Missouri, USA). Fatty acids were expressed as percentages of each individual fatty acid relative to the total of all fatty acids present in the sample. Fatty acid methyl esters were expressed as the percentage of total FAME.

Individual amino acid content in meat samples was determined according to the International Organization for Standardization [46]. A 0.5 g sample was measured twice in two separate 100 mL screw cap glass bottles. In the first, 5 mL performic acid was added and the bottle was refrigerated for one night. Then, 0.86 g sodium metabisulphite was added to decompose the remaining performic acid. To both bottles, 25 mL 6 M HCl was added and the bottles were placed in a 110 °C oven for 24 h. After that, the content of the bottles was washed in 200 mL volumetric flasks, the pH was set to 2.2 and the bottles were filled to the mark with pH 2.2 citrate buffer. The contents of the bottles were filtered and measured with an INGOS AAA-400 amino acid analyser (Prague, Czech Republic), on INGOS’s proprietary ion-exchange resin: OSTION LG ANB. The cysteine and methionine content were obtained from the oxidised sample (the first sample containing performic acid). The remaining amino acid contents were obtained from the second sample. The software used by the analyser is the freely available ChromUlan software (Prague, Czech Republic). As tryptophan is transformed by acidic hydrolysis into ammonium, it cannot be determined. The content of amino acids was expressed as mg/100 g of meat.

Determination of the contents of calcium, copper, iron, magnesium, manganese, potassium, sodium and zinc was done according to the method using atomic absorption spectrometry [47]. Namely, a 10-g sample was measured in a crucible and dried in an oven at 105 °C overnight, then transferred in a furnace and ashed at 500 °C for 8 h. The next day, 20 mL 6 M HCl was added to the sample and it was heated on a hot plate for 30 min. After cooling, the contents of the crucible were filtered into a 100 mL volumetric flask. The necessary dilutions for the measurement of the different elements were made from this solution and measured on a Solar M/atomic absorption spectrophotometer. Phosphorus content was measured from the sample in a 100 mL volumetric flask by adding molybdate-metavanadate reagent and extinction was measured with a spectrophotometer [48]. The results were expressed as mg/100 g of meat.

### 2.3. Statistical Analyses

Distribution and homogeneity of variance of samples was tested using Shapiro-Wilk test in SAS Software (Cary, NC, USA) [49]. Data that showed normal distribution were analysed using on-way ANOVA, while data that did not have normal distribution were tested using the Kruskal-Wallis test. Significance was tested at *p* < 0.05.

## 3. Results

### 3.1. Proximate Chemical Composition

The effect of sex and age on the proximate chemical composition of axis deer meat is presented in Table 1. No differences were observed between the analysed sex and age groups. Male and female axis deer had similar dry matter content, while adults had a higher (*p* > 0.05) dry matter content than sub-adult deer. Protein content was consistent between all analysed groups, whereas more differences were observed in fat content. Lower (*p* > 0.05) fat content was found in sub-adult males than females. Samples originating from adult male were also characterised by a lower (*p* > 0.05) ash content.

### 3.2. Fatty Acid Composition

Table 2 presents fatty acid composition and content of axis deer meat. The most abundant saturated fatty acids (SFA) were C16:0 and C18:0, accounting for more than 93% of all SFAs. However, SFAs accounted for less than 45% of the total fatty acids (FA) in the analysed samples. Between sex groups had very similar C16:0 contents, while it was lower (*p* > 0.05) for sub-adults than adults. The content of C18:0 was similar across analysed sex and age groups. Regarding monounsaturated fatty acids (MUFA), the most abundant was C18:1n9 (prox. 20%), while higher (*p* < 0.005) C18:1n-7 content was found in males than in females.

Analysed samples contained about 25% total MUFAs. Polyunsaturated fatty acids (PUFA) in the analysed samples accounted for 30–36% of FA with C18:2n6 as the most abundant PUFA. The sub-adult group had higher (*p* < 0.05) C18:3n3, C20:3n3, C20:5n3 and C22:6n3 content than the adult group. Analysed samples were also characterised by a high content of C20:4n6 and C22:5n3 PUFAs, and the sum of n-3 PUFAs was higher (*p* < 0.05) in sub-adults than in adults. A higher (*p* < 0.05) n-6/n-3 ratio was found in adults than in sub-adults (Table 2).

### 3.3. Amino Acid Content

The effect of sex and age on amino acid content of axis meat is shown in Table 3. The most abundant non-essential amino acids in both sex and age groups were glutamic and aspartic amino acid (3.90% and 2.20%, respectively), followed by alanine and glycine. Adult axis deer had a higher (*p* < 0.05) content of cysteine than sub-adults. The three most abundant essential amino acids in the present study were: lysine, leucine and arginine (1.92%; 1.70%; 1.38%, respectively), while threonine, valine and phenylalanine were represented each by 1%. Total essential amino acid content was 10.70%, whereas the content of non-essential amino acids was about 11.50%. Higher cysteine and methionine contents were found in the adult group, while females had a higher histidine content. The calculated essential and non-essential amino acid ratio was higher (*p* < 0.005) in females than in males and was lower than <1 for the analysed groups.

### 3.4. Mineral Content

Only a minor effect of sex and age was found on the mineral content of meat (Table 4). Very low concentrations (<1 mg/kg) of calcium (Ca) and manganese (Mn) were measured. Potassium (K) was the most abundant mineral in axis meat, followed by phosphorus (P), which was higher (*p* < 0.05) in younger individuals than the adult ones. Sodium (Na) content was similar for male and adult groups, whereas a similar Na content was found for the female and sub-adult groups. Magnesium (Mg) did not differ between groups (0.25–0.26 g/kg).

Micro-elements analysed in this study were manganese (Mn), iron (Fe), zinc (Zn) and copper (Cu) (Table 4). Iron (Fe) was the most abundant micro-element, with a higher (*p* > 0.05) content found in male and sub-adult groups than in female and adult groups. A higher (*p* > 0.05) content of zinc (Zn) was found in male and adult groups than in female and sub-adult groups. Copper (Cu) was less abundant, with a similar content in all groups (1.30%), and a very low (<1 mg/kg) Mn content was found in all groups.

## 4. Discussion

### 4.1. Proximate Chemical Composition

Meat is primarily a source of protein which is a less variable component than fat. Thus, the main differences in the proximate chemical composition between different species can be found in meat fat content. Because of health-related risk, the recommended dietary fat intake should be 20–35% and lean, low-fat meat has become more appreciated in the human diet [1]. In general, regarding proximate chemical composition, axis deer meat is characterised by a high-protein (>20%) and low-fat content (<2%) similar to the meat of other deer species [1]. Dry matter, protein and ash contents of axis deer meat were similar to those reported for other deer species, though major differences were observed in fat content [11,12,14,16,17,18,20].

Cawthorn et al. [20] and Švrčula et al. [12] reported higher fat content in both female and male fallow deer than in the present study. A lower fat content than in the present study was reported in male and female fallow deer by Piaskowska et al. [11] and Daszkiewicz et al. [16], and for male red deer [18]. Bureš et al. [14] found a lower fat content in the meat of male fallow and red deer, while Daszkiewicz et al. [9] reported a similar fat content in female, but lower content in male roe deer. A lower fat content was also found in male mouflon co-inhabiting the same area with axis deer [50]. In the present study, no significant effects of sex were found on meat proximate chemical composition, which corresponds to results reported elsewhere [17,20]. However, several studies have reported significant differences between male and female deer species [9,11,16]. It was documented that female animals in general, including ungulates, deposit more body and muscle fat than males. This is mainly due to physiologically related differences and energy needs during gestation [20,51]. As this is one of the first studies presenting proximate chemical composition in axis deer, the observed differences in regard to other deer species could be species related or the result of different methods applied for chemical analyses, location, age and diet.

No significant effect of age on proximate chemical composition of axis deer meat was found. However, Cygan-Szczegielniak and Janicki [8] reported that age affected dry matter and fat content of roe deer meat. Effect of age on fat content in fallow deer and wild red deer was reported by Volpelli et al. [7] and Lorenzo et al. [13]. Although deer meat is characterised by a low fat content, this was found to increase by age, sometimes even to unacceptably high levels [52]. An increase with age and lower meat fat content was observed for sub-adult axis deer, similar to reports for other deer species [7,8,13]. On the contrary, mouflon were reported to have a decrease of fat content with age [50].

### 4.2. Fatty Acid Composition

In addition to fat content, more interest has been placed on fat quality, i.e., fatty acid composition. Saturated fatty acids (especially C12:0, C14:0 and C16:0) are known to contribute to cardiovascular diseases and their intake should not exceed 10%. The most abundant SFA in meat is C16:0. Health-promoting benefits are associated with polyunsaturated fatty acids (PUFAs). Therefore, it is important to analyse and describe the fatty acid composition of meat originating from different species.

In the present study, C16:0 content in male and female axis deer meat was similar to that reported in male and female roe deer [9] and male fallow deer [14]. A higher C16:0 content in meat of male and female fallow deer than in the present study was reported by Daszkiewicz et al. [16] and Piaskowska et al. [11]. Several studies [12,14,17] reported a lower C16:0 content in the meat of male and female red and fallow deer. A lower C16:0 [50] was also reported for male mouflon. In the present study, lower SFA by sex was found than in Daszkiewicz et al. [9,16], Piaskowska et al. [11] and Švrčula et al. [12], but higher than for red deer by Bureš et al. [14] and Razmaite et al. [17]. Danszkiewicz et al. [9,16] and Piaskowska et al. [11] reported considerably lower PUFA for roe and fallow deer, whereas a similar PUFA was reported for red and fallow deer by Bureš et al. [14]. As in the present study, Razmaite et al. [17] and Švrčula et al. [12] reported a higher PUFA in female and male deer, while a higher PUFA in male than female deer was reported by Piaskoweska et al. [11] and Danszkiewicz et al. [9]. In male mouflon meat, a higher PUFA was reported than in the present study [50].

An increase of C16:0 and SFA by age found in this study corresponds to the results reported for red and fallow deer [7,13], however those studies reported a lower C16:0 and SFA content in all analysed age groups. Decrease of PUFA by age found in the present study corresponds to the results reported by Volpelli et al. [7] for fallow deer, although PUFA content was considerably higher than for axis deer. Contrarily, an increase of PUFA by age was found in red deer [13] and in mouflon [50].

In order to evaluate nutritional value of meat, the most often used ratios regarding fatty acids are PUFA/SFA (<4.0) and n-6/n-3 (>0.4). Both ratios for axis deer were favourable, with a higher (*p* > 0.05) n-6/n-3 for adult than for the sub-adult group. A significant increase of n-6/n-3 by age was also reported by Lorenzo et al. [13] and Kelava Ugarković and Ugarković [50].

In general, the fatty acid composition of axis deer meat is similar to other deer species. Axis meat is characterised by moderate SFA and high PUFA and favourable n-6/n-3 and P/S ratios. Differences in meat fatty acid composition between deer species are mainly due to diet composition and the quantity of supplementing feeding.

### 4.3. Amino Acid Content

Protein is the dominant component of meat dry matter and a valuable source of micronutrients i.e., amino acids. The nutritional value of meat protein depends on the presence or absence of non-essential and essential amino acids. As in the present study, a minor effect of sex and age on amino acid content was reported for red deer and springbok [13,53]. Cygan-Szczegielniak and Janicki [8] also reported a minor effect of sex on non-essential amino acids in roe deer meat, but a significant effect of essential amino acid content. Namely, the majority of analysed essential amino acids had a higher content in female than in male roe deer.

Glutamic and aspartic acid, alanine, lysine and leucine are the most abundant amino acids in game meat [52], and this corresponds to the results of this study. Non-essential amino acids (g/100 g meat) found in axis meat had a similar content to those reported by Lorenzo et al. [13] in wild red deer and by Hoffman et al. [53] in springbok meat. Analysing amino acid content in five ungulate game species (wild and farmed deer, roe deer, elk, wild boar), Strazdina et al. [5] reported a lower content of major non-essential amino acids than found in this study. Essential amino acids (g/100 g meat) found in axis deer were lower than reported by Lorenzo et al. [13], Strazdina et al. [5] and Hoffman et al. [53] for other deer species. However, for roe and maral deer, considerably higher non-essential and essential amino acid contents were reported than for axis deer [8,15]. Compared to domestic species (beef, pork, horse), it seems that axis deer has a lower content of essential and majority non-essential amino acids [54,55]. However, the observed differences can be the result of diet and different muscle samples. A 100-g serving of axis meat has one-third (prox. 10.6 g/100 g of meat) the daily recommended essential amino acid requirement (36 g/100 g of meat; FAO). This study contributes to better insight of the influence of sex and age on the composition of amino acids in deer meat as available data regarding this topic are scarce [52].

### 4.4. Mineral Content

Mineral content in the deer diet has a vital effect on their physiology and health. As axis deer are intermediate feeders, they graze and browse on numerous varieties of plants, and the resulting mineral composition is reflected in the meat.

Sex and age had a minor effect on the mineral content in axis deer meat. Similarly, a minor effect of sex and/or age on macro- and micro-mineral content was reported in wild red and fallow deer meat [13,20]. On the contrary, a significant effect of sex and/or age on trace element concentrations in deer and impala meat was reported by Dannenberger et al. [10] and Hoffman et al. [51].

In axis deer meat, the most abundant macro-minerals (g/kg) were P and K. This corresponds to the results for other deer species reported in earlier studies [13,20]. On the contrary, Zomborszky et al. [4] reported lower K, P and Na, but higher Ca and Mg content in red, fallow and roe deer from Hungary. Sodium (Na) content found in axis deer meat was similar to the meat of roe deer [20], but lower than wild red deer meat [13] and three other deer species [4]. Although Ca content in meat is low, in the present study it was considerably lower than in earlier studies [13,20]. Lorenzo et al. [13] and Dannenberg et al. [10] reported a similar Fe content for wild red deer and roe deer as found in axis deer meat, while Cawthorn et al. [20] reported higher Fe in fallow deer. Zinc (Zn) and copper (Cu) content of axis deer was similar to fallow deer [20], while a lower Zn and higher Cu content was reported for red deer [13]. Hungarian deer species [4] had a lower content of Fe, Zn and Cu than that found in this study. A similar content of manganese (Mn) reported for red and fallow deer correspond to the content found in this study (<1 mg/kg). Several studies reported mineral composition and content of antelope species. Higher content of P, K, Mg, Ca, Fe and Zn, but similar Na content was reported for impala [51], while half the P, K and Na content was reported for springbok meat [53]. Analysing the mineral content of internal organs of grazing red deer, Grace et al. [56] found potassium to be the most abundant mineral, whereas Vengušt and Vengušt [57] found phosphorus to be the dominant macro element in the liver of grazing fallow deer.

The observed variations in macro and micro-mineral content between different wild species are the result of different diet and geographical origin [52]. Namely, the content of minerals in soil and their availability for plants is reflected in meat. A very low Ca content found in axis deer can also be a result of lower Ca in water, and a calcium-salt licking block should be offered in order to eliminate Ca deficiency. Potential differences in mineral content between this and previous studies can also be due to different methods used to analyse mineral contents in meat [20].

## 5. Conclusions

This study is one of the first to examine in detail the nutrient composition in axis deer meat according to sex and age. Proximate chemical composition of axis deer meat corresponds to meat of other deer species (high-protein and low-fat) and no special considerations are needed regarding fatty acid composition (moderate SFA, high MUFA and PUFA). Axis meat can be considered a good source of essential amino acids and micro-minerals like iron and zinc. As minor differences were found between sex and age groups, it can be concluded that axis deer meat is in the range of the general characteristics of other game species, offering beneficial nutritional and health-related characteristics.

## Figures and Tables

**Table 1:** Proximate chemical composition of axis deer *longissimus thoracis*, as influenced by sex and age (mean ± SD).

Parameter (%)	Sex		*p*	Age		*p*	Overall Mean (*n* = 16)
	Male (*n* = 8)	Female (*n* = 8)		Sub-Adult (*n* = 8)	Adult (*n* = 8)		
Dry matter	26.52 ± 1.19	26.36 ± 0.97	0.919	25.30 ± 0.35	27.14 ± 1.19	0.262	26.45 ± 0.77
Protein	22.82 ± 0.21	22.73 ± 0.32	0.780	22.82 ± 0.41	22.77 ± 0.16	0.903	22.79 ± 0.18
Fat	1.28 ± 0.14	1.54 ± 0.31	0.530	1.09 ± 0.13	1.57 ± 0.61	0.300	1.39 ± 0.20
Ash	1.27 ± 0.04	1.30 ± 0.05	0.621	1.35 ± 0.02	1.24 ± 0.03	0.095	1.28 ± 0.03

**Table 2:** Fatty acid composition of axis deer *longissimus thoracis*, as influenced by sex and age (mean ± SD).

Fatty Acids (%)	Sex		*p*	Age		*p*	Overall Mean (*n* = 16)
	Male (*n* = 8)	Female (*n* = 8)		Subadult (*n* = 8)	Adult (*n* = 8)		
C12:0	0.05 ± 0.01	0.06 ± 0.01	0.903	0.05 ± 0.01	0.06 ± 0.01	0.198	0.05 ± 0.01
C14:0	1.75 ± 0.34	1.59 ± 0.44	0.787	1.09 ± 0.18	2.03 ± 0.37	0.083	1.68 ± 0.26
C15:0	0.32 ± 0.02	0.39 ± 0.06	0.216	0.29 ± 0.02	0.39 ± 0.04	0.132	0.35 ± 0.03
C16:0	23.81 ± 1.82	23.92 ± 2.94	0.973	20.27 ± 1.56	26.01 ± 2.12	0.078	23.86 ± 1.58
C17:0	0.51 ± 0.05	0.65 ± 0.10	0.229	0.50 ± 0.05	0.62 ± 0.08	0.310	0.57 ± 0.05
C18:0	18.40 ± 0.95	17.89 ± 1.81	0.794	17.66 ± 1.06	18.49 ± 1.36	0.675	18.18 ± 0.92
C20:0	0.23 ± 0.03	0.19 ± 0.03	0.376	0.20 ± 0.03	0.23 ± 0.03	0.522	0.22 ± 0.02
C22:0	0.06 ± 0.01	0.05 ± 0.01	0.388	0.06 ± 0.01	0.06 ± 0.01	0.976	0.06 ± 0.01
C14:1	0.13 ± 0.04	0.16 ± 0.05	0.646	0.09 ± 0.02	0.19 ± 0.04	0.090	0.15 ± 0.03
C16:1	2.27 ± 0.31	2.48 ± 0.49	0.709	1.83 ± 0.33	2.68 ± 0.36	0.132	2.36 ± 0.27
C18:1n9	20.38 ± 1.25	19.63 ± 1.69	0.720	19.01 ± 1.57	20.67 ± 1.29	0.435	20.05 ± 0.99
C18:1n7	3.04 ± 0.19	2.24 ± 0.13	0.005	2.78 ± 0.17	2.63 ± 0.23	0.677	2.69 ± 0.16
C20:1	0.13 ± 0.01	0.13 ± 0.03	0.964	0.15 ± 0.04	0.12 ± 0.01	0.268	0.13 ± 0.02
C18:2n6	16.37 ± 1.12	15.70 ± 1.23	0.693	15.31 ± 1.66	16.54 ± 0.86	0.474	16.08 ± 0.80
C18:3n6	0.04 ± 0.01	0.05 ± 0.01	0.350	0.06 ± 0.01	0.04 ± 0.01	0.236	0.04 ± 0.01
C18:3n3	1.54 ± 0.17	1.99 ± 0.39	0.265	2.30 ± 0.25	1.41 ± 0.22	0.021	1.74 ± 0.19
C20:2	0.11 ± 0.02	0.10 ± 0.02	0.661	0.13 ± 0.02	0.09 ± 0.02	0.197	0.11 ± 0.01
C20:3n6	0.33 ± 0.05	0.35 ± 0.08	0.776	0.42 ± 0.05	0.30 ± 0.06	0.171	0.34 ± 0.04
C20:4n6	8.46 ± 1.51	9.21 ± 2.33	0.783	10.41 ± 1.81	7.81 ± 1.74	0.343	8.78 ± 1.28
C20:3n3	0.06 ± 0.01	0.07 ± 0.01	0.446	0.09 ± 0.01	0.06 ± 0.01	0.003	0.07 ± 0.01
C20:5n3	1.44 ± 0.22	1.88 ± 0.44	0.361	2.24 ± 0.29	1.27 ± 0.27	0.034	1.63 ± 0.23
C22:5n3	3.74 ± 0.48	3.88 ± 0.79	0.881	4.76 ± 0.39	3.23 ± 0.58	0.082	3.80 ± 0.43
C22:6n3	0.15 ± 0.04	0.24 ± 0.07	0.297	0.35 ± 0.06	0.10 ± 0.02	0.001	0.19 ± 0.04
SFA	43.59 ± 2.15	42.47 ± 3.30	0.771	40.12 ± 2.49	44.89 ± 2.41	0.214	42.77 ± 2.49
MUFA	25.07 ± 1.09	24.06 ± 1.59	0.593	23.85 ± 1.83	25.10 ± 0.99	0.519	25.37 ± 1.08
PUFA	31.33 ± 2.96	33.47 ± 4.84	0.699	36.04 ± 4.07	30.08 ± 3.34	0.277	29.66 ± 3.44
n-3PUFA	6.95 ± 0.87	8.06 ± 1.67	0.536	9.73 ± 0.93	6.06 ± 1.05	0.032	7.43 ± 0.86
n-6PUFA	24.28 ± 2.34	25.31 ± 3.37	0.799	26.18 ± 3.45	23.86 ± 2.38	0.575	22.23 ± 2.67
n-6/n-3	3.90 ± 0.53	4.53 ± 1.19	0.612	2.74 ± 0.31	5.04 ± 0.82	0.050	3.08 ± 0.16
P/S	0.76 ± 0.12	0.87 ± 0.18	0.611	0.95 ± 0.17	0.73 ± 0.12	0.296	0.75 ± 0.12

SFA = ∑ of saturated fatty acids; MUFA = ∑ sum of monounsaturated fatty acid; PUFA = ∑ of polyunsaturated fatty acids; n-3 PUFA = ∑ of n-3 polyunsaturated fatty acids; n-6 PUFA= ∑ of monounsaturated fatty acids; n-6/n-3 = ratio between n-6 and n-3; P/S = ratio between PUFA and SFA.

**Table 3:** Amino acid composition and content of axis deer *longissimus thoracis*, as influenced by sex and age (mean ± SD).

Amino Acid (g/100 g Meat)	Sex		*p*	Age		*p*	Overall Mean (*n* = 16)
	Male (*n* = 8)	Female (*n* = 8)		Sub-Adult (*n* = 8)	Adult (*n* = 8)		
Non-essential							
Glutamic	3.95 ± 0.03	3.88 ± 0.07	0.303	3.91 ± 0.09	3.92 ± 0.02	0.825	3.92 ± 0.04
Aspartic	2.19 ± 0.03	2.20 ± 0.03	0.836	2.23 ± 0.04	2.18 ± 0.02	0.203	2.20 ± 0.02
Alanine	1.32 ± 0.02	1.29 ± 0.02	0.400	1.30 ± 0.03	1.31 ± 0.01	0.499	1.31 ± 0.01
Glycine	1.28 ± 0.04	1.24 ± 0.04	0.382	1.23 ± 0.04	1.28 ± 0.03	0.377	1.26 ± 0.03
Proline	1.03 ± 0.01	0.99 ± 0.02	0.075	0.99 ± 0.03	1.03 ± 0.01	0.175	1.01 ± 0.01
Serine	0.87 ± 0.01	0.87 ± 0.02	0.809	0.89 ± 0.02	0.86 ± 0.01	0.209	0.87 ± 0.01
Tyrosine	0.71 ± 0.01	0.72 ± 0.01	0.634	0.72 ± 0.01	0.71 ± 0.01	0.480	0.71 ± 0.01
Cysteine	0.20 ± 0.00	0.19 ± 0.01	0.127	0.18 ± 0.01	0.20 ± 0.00	0.035	0.19 ± 0.00
Essential							
Lysine	1.92 ± 0.02	1.93 ± 0.03	0.917	1.93 ± 0.03	1.92 ± 0.02	0.887	1.92 ± 0.02
Leucine	1.70 ± 0.02	1.71 ± 0.02	0.792	1.71 ± 0.02	1.70 ± 0.02	0.817	1.71 ± 0.01
Arginine	1.39 ± 0.01	1.36 ± 0.02	0.256	1.37 ± 0.03	1.39 ± 0.01	0.454	1.38 ± 0.01
Threonine	1.07 ± 0.01	1.02 ± 0.02	0.947	1.04 ± 0.02	1.00 ± 0.01	0.109	1.02 ± 0.01
Valine	1.05 ± 0.01	1.07 ± 0.01	0.356	1.07 ± 0.01	1.06 ± 0.02	0.476	1.06 ± 0.01
Phenylalanine	1.03 ± 0.02	1.06 ± 0.01	0.132	1.06 ± 0.01	1.03 ± 0.02	0.185	1.04 ± 0.01
Histidine	0.99 ± 0.03	1.06 ± 0.01	0.044	1.05 ± 0.02	0.99 ± 0.03	0.186	1.02 ± 0.02
Isoleucine	0.95 ± 0.01	0.95 ± 0.01	0.821	0.96 ± 0.01	0.95 ± 0.01	0.797	0.95 ± 0.01
Methionine	0.59 ± 0.01	0.57 ± 0.01	0.236	0.56 ± 0.01	0.59 ± 0.01	0.038	0.58 ± 0.01
∑EAA	10.63 ± 0.12	10.74 ± 0.14	0.610	10.75 ± 0.17	10.64 ± 0.12	0.608	10.68 ± 0.10
∑non-EAA	11.55 ± 0.10	11.37 ± 0.19	0.360	11.45 ± 0.25	11.49 ± 0.08	0.842	11.48 ± 0.10
EAA/non-EAA	0.92 ± 0.00	0.94 ± 0.01	0.002	0.94 ± 0.01	0.93 ± 0.01	0.260	0.93 ± 0.01

∑ EAA = sum of essential amino acids; ∑ non-essential = sum of non-essential amino acids; EAA/∑ non-EAA = ratio between essential and non-essential amino acids.

**Table 4:** Mineral composition and content of axis deer *longissimus thoracis*, as influenced by sex and age (mean ± SD).

Mineral Content	Sex		*p*	Age		*p*	Overall Mean (*n* = 16)
	Male (*n* = 8)	Female (*n* = 8)		Subadult (*n* = 8)	Adult (*n* = 8)		
Macro-minerals (g/kg)							
Calcium	<0.001	<0.001	-	<0.001	<0.001	-	<0.001
Magnesium	0.25 ± 0.04	0.26± 0.04	0.120	0.26 ± 0.04	0.25 ± 0.04	0.175	0.25 ± 0.04
Phosphorus	2.05 ± 0.03	2.09 ± 0.05	0.527	2.16 ± 0.04	2.01 ± 0.02	0.002	2.07 ± 0.03
Potassium	3.93 ± 0.30	3.68 ± 0.19	0.531	4.16 ± 0.48	3.62 ± 0.07	0.169	3.82 ± 0.19
Sodium	0.46 ± 0.04	0.51 ± 0.07	0.508	0.53 ± 0.08	0.46 ± 0.03	0.347	0.49 ± 0.05
Micro-minerals (mg/kg)							
Manganese	<1	<1	-	<1	<1	-	<1
Copper	1.34 ± 0.10	1.28 ± 0.12	0.742	1.38 ± 0.13	1.28 ± 0.09	0.520	1.35 ± 0.07
Zinc	22.88 ± 4.44	13.37 ± 0.99	0.085	15.51 ± 1.65	20.64 ± 4.26	0.384	18.72 ± 2.75
Iron	28.39 ± 4.42	24.72 ± 1.87	0.500	29.20 ± 6.98	25.34 ± 1.01	0.489	26.79 ± 2.59
